# Chemical basis of microbiome preference in the nematode *C. elegans*

**DOI:** 10.1038/s41598-024-51533-6

**Published:** 2024-01-16

**Authors:** Victor Z. Chai, Tiam Farajzadeh, Yufei Meng, Sokhna B. Lo, Tymmaa A. Asaed, Charles J. Taylor, Elizabeth E. Glater

**Affiliations:** 1 Department of Neuroscience, Pomona College, Claremont, CA USA; 2 Department of Chemistry, Pomona College, Claremont, CA USA

**Keywords:** Microbiology, Neuroscience

## Abstract

Animals are exposed to many microbes in their environment, some of which have been shown to colonize various tissues including the intestine. The composition of the intestinal microbiota affects many aspects of the host’s physiology and health. Despite this, very little is known about whether host behavior contributes to the colonization. We approach this question in the nematode *C. elegans*, which feeds on bacteria and also harbors an intestinal microbiome. We examined the behavior of *C. elegans* towards CeMbio, a simplified microbiome consisting of twelve strains that represent the bacteria found in the animal’s natural environment. We observed that *C. elegans* raised on *E. coli* shows a strong preference for three members of CeMbio (*Lelliottia amnigena* JUb66*, Enterobacter hormaechei* CEent1, and *Pantoea nemavictus* BIGb0393) compared to *E. coli*. Previously, these three bacterial strains have been shown to support faster *C. elegans* development time than *E. coli* OP50 and are low colonizers compared to eight other members of CeMbio. We then used gas chromatography coupled to mass spectrometry to identify that these three bacteria release isoamyl alcohol, a previously described *C. elegans* chemoattractant. We suggest that *C. elegans* seeks bacteria that release isoamyl alcohol and support faster growth.

## Introduction

The microbiome, particularly those microbes that reside in the intestine, play a critical role in their host’s physiology including nutrition, development, longevity and immunity^1^. Notably the microbiome composition and its changes have been shown to be associated with human neurological conditions, including Parkinson’s Disease, Autism Spectrum Disorder, depression, and anxiety^2,3^. Further, the composition of the intestinal microbiome is distinct from those that the animal is exposed to in its environment, indicating that some microbes are preferentially selected for colonization^4–6^. Previous studies have shown that the host’s genetic background can influence the composition of the gut microbiome^7,8^. However, the role of the host’s behavior has been explored little.

*Caenorhabditis elegans*, a free-living nematode, is an excellent organism for examining how a host interacts with microbes because it shows preferences for different bacterial strains^9–11^. Some bacteria provide nutritious food sources; while others resist digestion and colonize the intestine^7,8^. Chemosensory cues are the main way that *C. elegans* discriminates among different species of bacteria^12^. Moreover, it has long been known that *C. elegans* is attracted to volatile chemicals released by bacteria^13^, but the characteristics of non-pathogenic bacteria that release these chemicals are less understood. For example, does *C. elegans* prefer the odor of bacteria that can strongly colonize its intestine? Does *C. elegans* prefer the odor of more nutritious bacteria that support a faster development time from first stage larva (L1) to adult? Is there a chemical signature of nutritious bacteria?

To address these questions, we used CeMbio, a simplified microbiome containing 12 bacterial strains that represent the core species that likely comprise the natural *C. elegans* microbiome^6^. All 12 bacterial species that comprise CeMbio can support development of *C. elegans* as a monoculture and as a community and colonize the *C. elegans* intestine to varying levels. In addition, the bacterial species that comprise CeMbio have been initially characterized: the growth rate of *C. elegans* on these different bacterial species, the degree of bacterial colonization of the intestine, and their predicted metabolic pathways^6^. However, the behavioral responses of *C. elegans* to the odors of CeMbio bacteria have not been examined.

We set out to measure the preference of *C. elegans* for the odors of the twelve bacterial species that comprise CeMbio. We found that *C. elegans* raised on the standard laboratory food source, *E. coli* OP50, showed a strong preference for the odors of three of the twelve tested bacterial isolates over *E. coli* OP50. These three bacterial strains, *Enterobacter hormaechei* CEent1, *Lelliottia amnigena* JUb66*,* and *Pantoea nemavictus* BIGb0393, support a shorter development time than five of the CeMbio strains and *E. coli* OP50, and are low colonizers of the intestine and so are likely nutritious food sources. We then used gas chromatography coupled with mass spectrometry (GC–MS) to identify the major volatile chemicals released by the bacterial isolates and found that all three attractive isolates released isoamyl alcohol, a well-studied chemoattractant^13–15^.

## Results

### Olfactory preference behavior of *C. elegans* for CeMbio 

**Figure 1:**
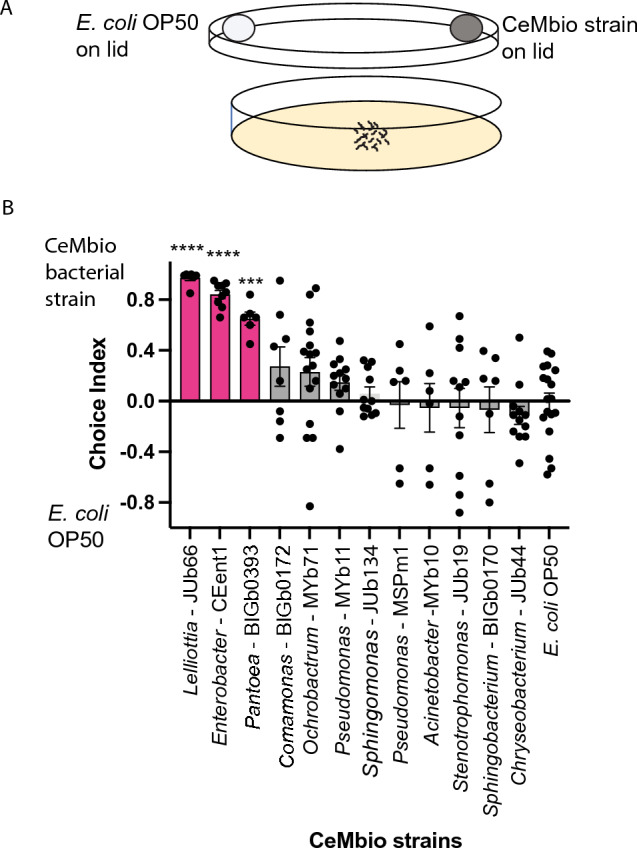
*C. elegans* preference for representative microbiome (CeMbio). (**A**) Cartoon of bacterial odor choice assay. Approximately 50–250 worms are placed on agar plate below two patches of bacteria on lid of plate. Animals approach the bacteria patches by olfactory chemotaxis. 1 μL of 1 M sodium azide was placed on NGM agar plate directly below each bacterial patch to immobilize worms when they reached the plate below each bacterial patch. Bacteria choice index is the number of worms under CeMbio bacteria minus number of worms under *E. coli* OP50 divided by sum of number of worms under CeMbio bacteria and under *E. coli* OP50. (**B**) Wildtype (N2) showed a significant preference for three of the CeMbio bacteria (shown in pink). ****p < 0.0001, ***p < 0.001, ANOVA with Dunnett compared to choice index between two patches of *E. coli* OP50 bacteria (last bar), n ≥ 6 assays.

To determine the preference of *C. elegans* for CeMbio bacterial species based only on volatile chemicals, we used a bacterial odor choice assay in which worms raised on *E. coli* OP50 are given a choice between the CeMbio bacteria and *E. coli* OP50, placed on the petri dish lid^16^. In this assay, where *C. elegans* only uses volatile chemical cues released by the bacteria to discriminate among the two bacterial species, *C. elegans* showed a significant preference for three bacterial strains, *Lelliottia amnigena* JUb66, *Enterobacter hormaechei* CEent1, and *Pantoea nemavictus* BIGb0393 over *E. coli* OP50 (Fig. 1). To our knowledge, this is the first time the attractiveness of JUb66 and CEent1 has been demonstrated. It has previously been shown that *Pantoea nemavictus* BIGb0393 and other *Pantoea *sp. are attractive to *C. elegans* over *E. coli * OP50 in bacterial choice assays^17^. In addition, consumption of *Enterobacter hormaechei* CEent1 has been shown to provide immune-protective effects in *C. elegans*^6^. Two of the preferred strains (JUb66 and CEent1) represent the most abundant family of bacteria, *Enterobacteriaceae*, found in the natural environment of *C. elegans*^6^. Until recently *Pantoea* BIGb0393 was thought to belong to the *Enterobacteriaceae* family, but now the genus *Pantoea* is classified as part of the *Erwiniaceae* family which is closely related to the *Enterobacteriaceae* family^18^.

*C. elegans* showed no preference in choice assays between the remaining nine CeMbio strains and *E. coli* OP50 (Fig. 1). The choice index for these bacterial strains vs. *E. coli* OP50 did not differ significantly from the choice index between two patches of *E. coli* OP50. We hypothesize that this is because neither the CeMbio strain or *E. coli* OP50 are releasing sufficient amounts of attractive or repulsive volatile chemicals under these low nutrient assay conditions to produce a consistent preference. Therefore, the worms are likely not using the volatile cues released by the bacteria to determine where to go on the plate, but rather they are responding to other cues on the plate that are difficult to control and vary from assay to assay such as slight moisture, thermal, or air flow gradients. We address this hypothesis by identifying volatile chemicals released by the bacteria in the next section.

### Identification of volatile chemicals released by CeMbio

Although *C. elegans* has been shown to exhibit innate preferences for the odor of different bacterial strains, the chemical basis of this discrimination is less well-understood. We hypothesized that the preferred strains would release chemicals that are attractive to *C. elegans* and that the no-preference strains would release neutral or non-detectable chemicals. We used gas chromatography-mass spectrometry (GC–MS) to identify the volatile chemicals present in the headspace of the twelve CeMbio bacterial strains and *E. coli* OP50. The term headspace refers to the volume of air above the bacterial sample.

**Figure 2:**
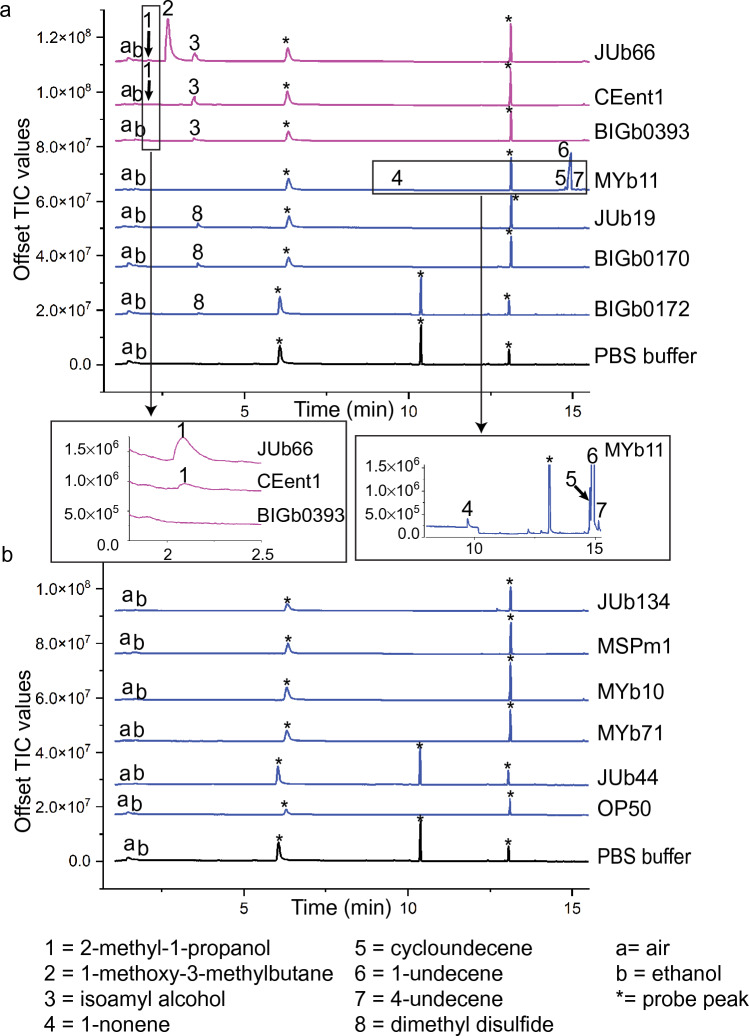
Gas chromatography–mass spectrometry of headspace of CeMbio bacterial strains. Overnight liquid cultures of bacteria were spotted on NGM agar plates (OD_600_ = 10) and incubated for 1 h, then NGM agar squares with bacterial suspension were placed inside a GC–MS glass vial for 24 h. One asterisk indicates “probe peaks,” i.e. volatile cyclic siloxanes released by the HiSorb probe. Peaks were identified tentatively with NIST 11 (National Institute of Standards and Technology) mass spectral library and confirmed with known standards except for cycloundecene and 4-undecene which could not be obtained. All bacterial samples were analyzed two or more times on different days. (**A**) Bacterial strains which volatile organic compounds were detected. Preferred strains are shown in pink; non-preferred strains are shown in blue. (**B**) Bacterial strains where no volatile organic compounds were detected.

**Figure 3:**
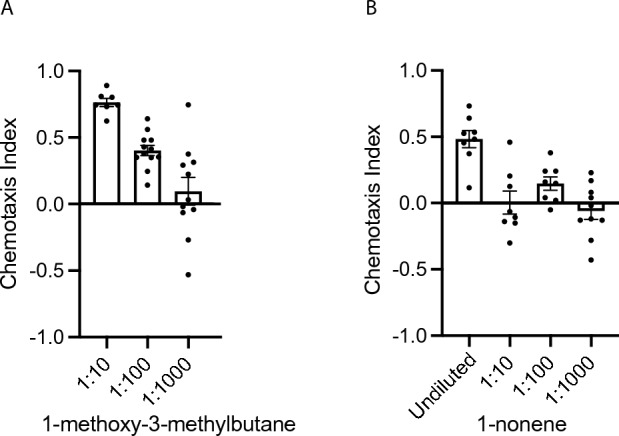
Chemotaxis to volatile organic compound released by attractive CeMbio strains. (**A**) 1-methoxy-3-methylbutane (shown to be released by *Lelliottia* JUb66) and diluted in ethanol. (**B**) 1-nonene (shown to be released by *Pseudomonas lurida* MYb11) and diluted in ethanol. n ≥ 6 assays conducted on at least two different days. Error bars represent SEM.

The headspace of the three attractive strains (*Lelliottia* JUb66, *Enterobacter* CEent1, and *Pantoea* BIGb0393) contained isoamyl alcohol, a volatile chemical that has been shown to be attractive to *C. elegans* in numerous studies at a broad range of concentrations from undiluted to 10^−4^ dilution^13–15^ (Fig. 2 and Table 1). In a previous study of volatile chemicals released by bacterial isolates found in the natural environment of *C. elegans* (but not CeMbio strains), isoamyl alcohol was also found to be released by the four most preferred isolates (*Alcaligenes *sp. JUb4, *Providencia* sp. JUb5, *Providencia* sp. JUb39, and *Flavobacteria* sp. JUb43)^16^. In comparison, nine other CeMbio strains and *E. coli* OP50 did not release isoamyl alcohol (Fig. 2). In addition to isoamyl alcohol, JUb66 also released 2-methyl-1-propanol and 1-methoxy-3-methylbutane. 2-methyl-1-propanol has been shown to be attractive to *C. elegans* in chemotaxis assays^13^ and was also shown to be released by other preferred bacterial strains (*Alcaligenes* sp. JUb4, *Providencia* sp. JUb39, and *Flavobacteria* sp. JUb43) found in the natural environment of *C. elegans*^16^. Because the attractiveness of 1-methoxy-3-methylbutane has not been previously tested, we tested the attractiveness of 1-methoxy-3-methylbutane in chemotaxis assays and found it to be attractive (Fig. 3). CEent1 released isoamyl alcohol and 2-methyl-1-propanol. BIGb0393 only released isoamyl alcohol.

**Table 1:** Summary of bacterial strains and volatile organic compounds.

Volatile organic compound	Retention time (min)	Figure 2 label	JUb66	CEent1	BIGb0393	MYb11	JUb19	BIGb0170	BIGb0172	*C. elegans* chemotaxis to pure chemical
2-Methyl-1-propanol	2.10	1	+	+						Attractive^13^
1-Methoxy-3-methylbutane	2.69	2	+++							Attractive (this study)
Isoamyl alcohol	3.48	3	++	++	+					Attractive^13^
Dimethyl disulfide	3.61	8					+	+	+	Neutral or weakly attractive^20–22^
1-Nonene	9.72	4				+				Weakly attractive (this study)
Cycloundecene	14.79	5				+				Unknown
1-Undecene	14.95	6				+++				Repulsive^19^
4-Undecene	15.14	7				+				Unknown

+++ indicates high abundance VOCs with total ion chromatogram (TIC) peak greater than 6.0 × 10^8^; ++ indicates medium abundant VOCs with TIC between 1.0 × 10^8^ and 2.0 × 10^8^; + indicates low abundant VOC with TIC between 8.0 × 10^6^ and 8.0 × 10^7^. All chemical identifications confirmed with chemical standards except cycloundecene and 4-undecene because standards could not be obtained. No VOCs were detected from JUb134, MSPm1, MYb10, MYb71, JUb44, and OP50 (not included in table).

*C. elegans* showed no preference for *Pseudomonas lurida* MYb11 over *E. coli* OP50 (Fig. 1). MYb11 was found to release a relatively high amount of 1-undecene and low amounts of 1-nonene, as well as cycloundecene and 4-undecene although the latter two compounds could not be verified because chemical standards could not be obtained (Fig. 2). 1-undecene was not shown to be released by any other CeMbio strains, but has been found to be released by the pathogenic bacterium *Pseudomonas aeruginosa*^19^. *C. elegans* shows repulsion to 1-undecene at high concentrations and weak repulsion or neutral responses at lower concentrations^19^. Because the response of *C. elegans* to 1-nonene has not been previously reported, we tested *C. elegans* response in chemotaxis assays. We found that the response to 1-nonene was attractive when undiluted and neutral at lower concentrations. The two additional chemicals, cycloundecene and 4-undecene, could not be tested because they could not be acquired. We suggest that *C. elegans* displays no preference between MYb11 and *E. coli* OP50 because MYb11 does not release sufficient levels of 1-undecene to be repulsive or because the other volatile chemicals suppress the repulsiveness of 1-undecene.

Three bacterial strains (*Stenotrophomonas indicatrix* JUb19, *Sphingobacterium multivorum* BIGb0170, and *Comamonas piscis* BIGb0172) were found to release the compound dimethyl disulfide (Fig. 2). *C. elegans* did not show a preference for any of these strains. Dimethyl disulfide has previously been found to be moderately attractive or neutral in *C. elegans* chemotaxis assays^20–22^.

No volatile chemicals were detected in the headspace of five of the CeMbio strains (*Ochrobactrum vermis* MYb71, *Acinetobacter guillouiae* MYb10, *Pseudomonas berkeleyensis* MSPm1, *Sphingomonas molluscorum* JUb134, and *Chryseobacterium scophthalmum* JUb44) (Fig. 2). In addition, *E. coli* OP50 also did not release any detectable volatile chemicals (Fig. 2). Consistent with the lack of detectable volatile chemicals, *C. elegans* did not show a significant preference for these five strains over *E. coli* OP50. We hypothesize that these bacterial strains release non-detectable amounts of volatile chemicals because they are not metabolically active in the bacterial growth conditions used in these experiments.

## Discussion

We examined the odor preference of *C. elegans* for different bacterial species that comprise the simplified representative microbiome of *C. elegans*, CeMbio. We found that *C. elegans* showed a significant preference for three strains (*Lelliottia* JUb66, *Enterobacter* CEent1, and *Pantoea* BIGb0393) over *E. coli* OP50 and that all three of these strains released isoamyl alcohol, a known *C. elegans* chemoattractant.

Our study shows that *C. elegans* prefers three of the 12 CeMbio bacteria compared to the standard laboratory food source *E. coli* OP50 strain. Previous studies examining the individual strains that comprise CeMbio identified several characteristics of these bacteria^6^. First, we compared bacterial preference to developmental time. Nine CeMbio strains resulted in a faster development time compared to *E. coli* OP50, while two strains (*Sphingobacterium* BIGb0170 and *Chryseobacterium* JUb44) resulted in a slower time and one strain had a similar time to *E. coli* OP50. *C. elegans* showed a strong preference for *Pantoea nemavictus* BIGb0393 which resulted in the second greatest percentage of adults at 52 h post L1^6^. *Lelliottia amnigena* JUb66 supported a developmental time above the median for all CeMbio strains and *Enterobacter hormaechei* CEent1 supported a developmental time just below the median. *C. elegans* did not show a preference for several of the species that supported a growth time shorter than the median (*Pseudomonas lurida* MYb11, *Sphingomonas molluscorum* JUb134, *Pseudomonas berkeleyensis* MSPm1, *Stenotrophomonas* JUb19, *Comamonas* BIGb0172). These results suggest that based on volatile cues *C. elegans* prefers some, but not all, bacterial strains that confer a faster growth time. Overall, there is a low correlation between development time and bacteria choice index in this study (linear regression r^2^ = 0.23). This is consistent with previous study of bacterial strains found in the natural environment of *C. elegans*, but not part of CeMbio, where a strong correlation (r^2^ = 0.52) was not found between bacterial preference and development time^16^.

We next examined the correlation between behavioral preference for bacteria and the level of bacterial colonization of the intestine. Dirksen et al. categorized the CeMbio bacterial strains for their ability to colonize individually the intestine after 72 and 120 h into three groups: low colonizers, medium colonizers, and high colonizers. It is of note that the bacterial strains for which *C. elegans* showed the highest preference were all low colonizing strains (*Lelliottia amnigena* JUb66, *Enterobacter hormaechei* CEent1, and *Pantoea nemavictus* BIGb0393). The other low colonizing bacteria was *Acinetobacter guillouiae* MYb10 for which *C. elegans* showed no preference over *E. coli* OP50. When *C. elegans* is cultured on the community of all twelve strains of CeMbio, these preferred strains are also low colonizers of *C. elegans* intestine^6^. Therefore, based on olfactory cues, *C. elegans* prefers low colonizing bacterial strains. Low colonizing bacterial strains may be preferred because they provide more nutrition to *C. elegans*.

In addition, Dirksen et al. analyzed the predicted number of metabolic pathways present each bacterial strain from genomic data. The three preferred strains have the three highest number of metabolic pathways of all the CeMbio strains. *Enterobacter* CEent1 has the highest number of metabolic pathways, 386, while *Chryseobacterium* JUb44 has the fewest, 186 pathways. The high number of metabolic pathways likely indicates that the preferred bacterial strains can be metabolically active in a wide variety of environments which provide different nutrient sources for growth. Specifically, a well-studied metabolic pathway that produces isoamyl alcohol as a byproduct is the catabolism of leucine by the Ehrlich pathway in *Saccharomyces cerevisiae*^23^. Based on protein predictions from genomic sequence data^6^, the enzymes that comprise this pathway are not present in the preferred bacterial strains. Further research is required to determine the metabolic pathway or pathways in each of the preferred strains that result in the release of isoamyl alcohol.

In addition to the preferred strains having the highest number of metabolic pathways, two of the attractive strains, *Lelliottia* JUb66 and *Enterobacter* CEent1, belong to the bacterial taxa that is found most often in the natural habitat of *C. elegans*. Each CeMbio strain was chosen as a representative of the most abundant bacterial taxa found in different natural environments of *C. elegans*, and *Lelliottia* JUb66 and *Enterobacter* CEent1, represent the most abundant bacterial taxa of all the CeMbio strains (operational taxonomic unit, or OTU1)^6^. A likely hypothesis is that *C. elegans* likely coexists with this bacterial taxon most often because they are most attracted to them. In the future, it would be interesting to examine other bacterial isolates belonging to this taxon to determine if they are also attractive.

Taken together, in this study, *C. elegans* showed olfactory preference for three CeMbio strains (*Lelliottia* JUb66, *Enterobacter* CEent1, and *Pantoea* BIGb0393). These strains are likely nutritional food sources, low colonizers of the intestines, and have the highest number of metabolic pathways. In addition, two of the attractive strains, *Lelliottia* JUb66 and *Enterobacter* CEent1 represent the most abundant bacterial taxa of all CeMbio strains. *C. elegans* is found naturally in bacterial-rich environments, particularly rotting fruit and compost heaps, shows innate preferences for different species of bacteria, and can detect and recognize volatile chemicals released from bacteria^12,24^. However, only recently, the chemical cues released by bacteria that *C. elegans* prefers have begun to be defined^16,19–21,25–28^. In this study and a previous study, isoamyl alcohol was found to be released by preferred bacterial strains. In a previous study, bacteria found in the natural environment but not part of CeMbio, four of the six attractive isolates (*Alcaligenes* JUb4, *Providencia* JUb5, *Providencia* JUb39, and *Flavobacteria* JUb43) were also found to release isoamyl alcohol^16^. The robust attractive response of *C. elegans* to isoamyl alcohol from undiluted to 10^−4^ dilution is well-studied^13–15^. The next question is to begin to understand the basis of preference or why *C. elegans* is attracted to some volatile chemicals released by bacteria and not by others. Perhaps, isoamyl alcohol often indicates an actively growing bacteria and an actively growing bacteria is likely to be a nutritious food source for *C. elegans*.

In the future, it would be interesting to examine other factors that affect *C. elegans* olfactory preferences for bacterial strains. Culturing bacteria in different defined media with different nutrient sources would likely result in utilization of different bacterial metabolic pathways and volatile profiles which may change olfactory preferences of *C. elegans*. For example, in this study *C. elegans* showed no preference for *Pseudomonas* MSPm1 or *Pseudomonas* MYb11. However, in another study *C. elegans* was found to be attracted to MSPm1 over *E. coli* HB101 and to be repulsed by MYb11^29^. In this other study, bacteria were resuspended in LB rather than PBS as in the current study. The LB provided more nutrients so that the bacteria likely released more and/or different volatile chemicals that mediated attraction or repulsion by *C. elegans*.

In addition to examining the effects of different nutrient sources to support bacterial growth, it would be interesting to examine olfactory responses to different mixtures of CeMbio strains which may result in different volatile chemicals being produced because the bacteria can use metabolites produced by each other to grow. For example, co-cultures of MYb71 and MYb11 produce different compounds compared with individual cultures of the two species^30^. The attractiveness and volatile profile of this combination of bacterial strains as well as other mixtures of CeMbio strains can be examined.

Overall, a goal of future work would be to define the volatile cues that attract *C. elegans* to bacterial strains under different bacterial growth conditions. The next step is to determine if there are consistent characteristics of the preferred bacteria that these cues signify, such as an indication of actively growing bacteria. This work contributes to a foundation for future work to understand better how *C. elegans* behavior affects interactions with its microbiome.

## Methods

### *C. elegans* and bacterial strains

*C. elegans* were grown and maintained under standard conditions at 20 °C on Nematode Growth Media (NGM). N2 worms were grown on NGM plates seeded with *E. coli* OP50. CeMbio bacterial strains were provided by *C. elegans* Genetics Center (CGC) which is funded by NIH Office of Research Infrastructure Programs (P40 OD010440): *Enterobacter hormaechei* CEent1, *Lelliottia amnigena* JUb66, *Acinetobacter guillouiae* MYb10, *Sphingomonas molluscorum* JUb134, *Stenotrophomonas indicatrix* JUb19, *Pseudomonas lurida* MYb11, *Pseudomonas berkeleyensis* MSPm1, *Comamonas piscis* BIGb0172, *Pantoea nemavictus* BIGb0393, *Ochrobactrum vermis* MYb71, *Sphingobacterium multivorum* BIGb0170, and *Chryseobacterium scophthalmum* JUb44.

### Bacterial odor choice assay

The bacterial odor choice assay which measures olfactory preference for bacteria was modified from Worthy et al. in order to follow the protocol used in Dirksen et al. for preparing CeMbio bacteria. Briefly, CeMbio bacterial strains and *E. coli* OP50 were grown overnight (except JUb134 was grown for 48 h because it grows more slowly than other CeMbio strains) in Luria Broth (LB) at 25 °C. 25 μL of each bacterial suspension (OD_600_ = 10) in PBS was spotted onto NGM plates and incubated for 24 h at room temperature. Then each agar square containing 25 μL bacteria patch was extracted using a sterile metal spatula. An NGM agar square with CeMbio bacterial isolate and an NGM agar square with *E. coli* OP50 were placed on opposite sides of a petri dish lid. 1 μL of 1 M sodium azide was pipetted on NGM agar directly below bacterial patch on lid to immobilize worms. Adult animals were washed three times in S-basal buffer, 50 to 250 were placed in the center of the NGM plate, equidistant from the two bacterial patches. Animals were allowed to move freely for 1 h and then were counted. The bacterial choice index is the number of worms on the CeMbio strain minus the number of worms on *E. coli* OP50 divided by the number of worms underneath both bacterial patches.

### Gas chromatography–mass spectrometry

Bacteria were prepared for GC–MS analysis in a similar method as described previously^16^. Bacteria were grown overnight in LB at 25 °C, centrifuged, and then resuspended in PBS at an OD_600_ = 10. Two NGM plates were prepared each with 9 spots of 25 μL of bacterial suspension. For the controls, 25 μL of PBS without bacteria was spotted on NGM plate. Plates were incubated for 1 h at 20 °C. Then 18 squares of the NGM agar with 25 μL bacteria suspension were placed in a GC–MS glass vial for 24 h at 20 °C. Headspace samples were collected using Markes PDMS-coated HiSorb probes and were analyzed by Thermal Desorption (TD) GC–MS using the Agilent 6890 GC System equipped with a Markes Unity II Thermal Desorption System on the GC inlet, a Restek, Rtx-5 column, and Agilent 5973 Mass Selective Detector. The thermal desorption sampling method was used rather than a solid-phase microextraction (SPME) fiber to increase sensitivity^31^. The temperature program was hold 8 min at 35 °C, increased to 130 °C at a rate of 10 °C/min, hold 5 min at 130 °C then increased to 300 °C at a rate of 15 °C/min, and hold at 300 °C for 1 min. MS ranged from m/z 30 to 550 in full scan mode. VOCs were identified with the NIST 11 (National Institute of Standards and Technology) mass spectral library and pure chemical standards run following the same parameters as for bacterial samples for all chemicals except for cycloundecene and 4-undecene which could not be obtained. Samples were prepared for analysis in duplicate on different days from a single stock of each bacterium and PBS control samples were run immediately before or after each bacterial sample.

### Chemotaxis assays

Chemotaxis assays were performed using 10 cm square chemotaxis plates as described^32^. In brief, assay agar was 2% agar, 1 mM MgSO_4_, 1 mM CaCl_2_, 5 mM phosphate buffer [pH 6.0]. Chemical dilutions were in ethanol at the concentrations indicated in figure legends. 2 μL of diluted chemical was pipetted on one side of the plate, 2 μL of ethanol on the other side, and 2 μL of 1 M sodium azide on both sides to anaesthetize animals that reached odor or ethanol sources. Adult animals were washed two times in S-basal buffer and one time in water, 50–200 animals were placed at the center of chemotaxis plate, plate was covered with lid, and the distribution of animals counted after 1 h.

### Statistical analysis

Means represent data pooled from assays run on at least two different days with at least 6 replicates. Error bars in all figures are standard error of means. The data were analyzed using statistics described in figure legends with GraphPad Prism v9.5 for Mac (GraphPad Software, San Diego California USA) or Microsoft Excel.

## Data Availability

The datasets generated and analyzed in this publication are available from the corresponding author upon request.
